# Advances in Implant-Based Breast Reconstruction in the Setting of Post-Mastectomy Radiotherapy

**DOI:** 10.1007/s00266-026-06219-x

**Published:** 2026-09-11

**Authors:** Qingyu Wang, Bingyue Li, Siyu Chen, Jun Wang

**Affiliations:** Department of Radiation Oncology, Hebei Clinical Research Center for Radiation Oncology, The Fourth Hospital of Hebei Medical University, Shijiazhuang, China

**Keywords:** Implant-based breast reconstruction, Radiotherapy, Two-stage breast reconstruction, Post-mastectomy radiation therapy (PMRT), Breast cancer

## Abstract

**Background:**

Breast reconstruction and post-mastectomy radiation therapy (PMRT) have become integral components of comprehensive breast cancer management. The coordination of radiation therapy and reconstruction must balance long-term oncologic safety with optimal aesthetic outcomes.

**Objective:**

This narrative review aims to evaluate and synthesize the latest advancements in the integration of implant-based breast reconstruction and radiotherapy. Specifically, it seeks to clarify the impact of reconstructive timing, material selection, anatomical placement, and advanced radiation techniques on clinical outcomes. The primary endpoint was defined as the incidence of treatment-related complications, and secondary endpoints included long-term aesthetic outcomes and oncologic safety.

**Methods:**

We systematically screened and synthesized the existing literature by searching core databases, including PubMed, Embase, and Web of Science.

**Results and Conclusion:**

Currently, the integration of implant-based breast reconstruction with radiotherapy remains an active area of investigation, with consensus lacking on many key aspects. The integration of implant-based reconstruction and radiotherapy necessitates multidisciplinary collaboration to develop individualized treatment strategies. Future prospective studies are warranted to optimize clinical pathways.

**No Level Assigned:**

This journal requires that authors assign a level of evidence to each submission to which Evidence-Based Medicine rankings are applicable. This excludes Review Articles, Book Reviews, and manuscripts that concern Basic Science, Animal Studies, Cadaver Studies, and Experimental Studies. For a full description of these Evidence-Based Medicine ratings, please refer to the Table of Contents or the online Instructions to Authors www.springer.com/00266

**Supplementary Information:**

The online version contains supplementary material available at https://doi.org/10.1007/s00266-026-06219-x.

## Introduction

Breast cancer ranks as one of the most common malignancies, accounting for approximately 11.6% of all cancer cases worldwide. Although nearly 99% of patients are female, its incidence rate in the entire population is second only to that of lung cancer [1, 2]. Post-mastectomy radiation therapy (PMRT) and breast reconstruction are essential components of multimodal treatment.

Breast reconstruction can be broadly categorized by the material used: autologous tissue, implants, or a combination of both [3]. A significant benefit of autologous breast reconstruction is the utilization of the patient’s own tissues, which substantially reduces the risk of immunogenic rejection associated with foreign materials. However, this approach is technically demanding, requires prolonged surgical time, and results in donor-site scarring, posing potential physical and psychological burdens. Consequently, for patients with a slender body habitus and limited donor tissue or for elderly patients unable to tolerate lengthy procedures, implant-based reconstruction represents a preferable alternative. Choosing implant-based reconstruction initially also preserves the option for future conversion to autologous techniques. For patients with insufficient autologous tissue volume for whom an implant alone cannot achieve adequate reconstruction, a combined approach is necessary. Among all breast reconstruction modalities, implant-based reconstruction is the most widely utilized.

Capsular contracture and implant malposition are well-established complications of breast reconstruction, with PMRT being a recognized contributing factor [4, 5]. The incorporation of breast reconstruction, while beneficial for patient quality of life, introduces challenges for radiation oncologists. The altered breast contour and volume may complicate both radiation treatment planning and scheduling, thereby adding complexity to the therapeutic process [6, 7].

Two international multidisciplinary consensus statements have established a framework and management guidance for breast reconstruction and radiotherapy, encompassing comprehensive considerations for both autologous and implant-based approaches, and providing evidence-based decision-making support and standardized pathways for clinical practice [8, 9]. Beyond these consensus statements, this review focuses specifically on the integration of radiotherapy and implant-based breast reconstruction, delving into the intricacies of their coordination. The primary endpoint was defined as the incidence of treatment-related complications, and secondary endpoints included long-term aesthetic outcomes and oncologic safety. We synthesize evidence on several key variables that have not been thoroughly explored, including the impact of different implant materials, the use of bolus, and chest wall boost irradiation. Additionally, we discuss preliminary clinical evidence for the application of proton therapy in this setting. Determining how to optimally coordinate radiotherapy with implant-based breast reconstruction represents a critical clinical and research priority. This review summarizes and discusses recent advances regarding the timing of reconstruction relative to PMRT, the influence of implant materials and placement, and specific radiotherapy techniques for patients with implants. The main findings and outcomes of the included key studies are summarized in Table 1.

**Table 1:** A summary of the main findings and outcomes of the included key studies

Category	Study	Study design	Sample size	Study endpoint	Key findings and outcomes
Reconstruction type	Matar et al. [12]	Meta-analysis	6 studies	Complications	No significant difference in complication rates was observed between immediate and delayed reconstruction. However, not all patients underwent implant-based reconstruction.
	Manyam et al. [13]	Retrospective study	*n*=77	Reconstruction failure, complications	The rate of complications requiring reoperation (38% vs. 70%) and reconstruction failure rate (22% vs. 56%) were both lower in the immediate reconstruction group than in the delayed reconstruction group. Infection may be the primary cause of reconstruction failure.
The timing of radiotherapy intervention	Cordeiro et al. [17]	Retrospective study	*n*=304	Reconstruction failure, complications, aesthetic results	The tissue expander radiation group had a higher reconstruction failure rate than the permanent implant radiation group (32% vs. 16.4%, *p*<0.01), but better aesthetic outcomes (75.0% vs. 67.6%, *p*<0.01) and lower grade IV capsular contracture (*p*<0.01).
	Lee et al. [18]	Meta-analysis	8 studies(*n*=899)	Reconstruction failure, complications	Compared with the implant radiation group, the tissue expander radiation group showed a trend toward higher risks of reconstruction failure and major complications requiring reoperation (not statistically significant), but a significantly lower risk of severe capsular contracture (*P*<0.001).
	Cagli et al. [19]	Retrospective study	*n*=183	Reconstruction failure, complications	Radiation delivered to the tissue expander was associated with a higher risk of reconstruction failure (OR = 2.77), whereas radiation to the permanent implant correlated with a higher likelihood of severe capsular contracture (OR = 2.7).
	Ricci et al. [20]	Meta-analysis	20 studies(*n*=2348)	Reconstruction failure, complications	Compared with irradiation to the permanent implant, irradiation to the tissue expander resulted in a higher reconstruction failure rate (20% vs. 13.4%, RR = 2.33, *P* = 0.0083) but a lower capsular contracture rate (24.5% vs. 49.4%, RR = 0.53, *P* = 0.083).
	Guo et al. [21]	Meta-analysis	11 studies(*n*=1447)	Reconstruction failure, complications	The risk of capsular contracture in the irradiated tissue expander group was significantly lower (RR 0.47; 95% CI: 0.29–0.78; *p*=0.003), and the incidence of other complications was comparable between the two groups. In contrast, the reconstruction failure rate was relatively lower in the irradiated permanent implant group (RR 1.71; 95% CI, 1.06–2.75; *p*=0.03).
	Woo et al. [22]	Retrospective study	*n*=49	Complications	Patients in the deflated group (*n*=20) had significantly more overall complications than the inflated group (*n*=29) (65% vs. 6.9%; *p*<0.001). Specific adverse events, including hematoma (40% vs. 0%; *p*<0.001) and skin fibrosis/contracture (25% vs. 3.4%; *p*=0.035), were also significantly more common in the deflated group.
	Peled et al. [16]	Retrospective study	*n*=88	Reconstruction failure	The implant removal rate was 22.4% in the <6-month post-radiotherapy exchange group and 7.7% in the ≥6-month group (*p*=0.036).
	Lentz et al. [23]	Retrospective study	*n*=34	Complications	When stratified by a 4-month threshold for expander-to-implant exchange after radiotherapy, the shorter-interval group had a higher incidence of infection (30% vs. 14.29%). In contrast, capsular contracture was more common in the longer-interval group (21.43% vs. 5.00%).
Reconstruction materials and radiotherapy	Cordeiro et al. [28]	Prospective study	*n*=3546	Incidence of BIA-ALCL	Long-term follow-up of 3,540 patients with textured implants revealed that 10 patients (10/3,540) developed BIA-ALCL after a median exposure of 11.5 years.
	Park et al. [31]	Retrospective study	*n*=26	Complications	Although the complication rate in the ADM group was lower than that in the control group without reaching statistical significance, predictive factor analysis revealed that the occurrence of complications was associated with the reconstruction type (ADM group vs. non-ADM group, *p* = 0.015)
	Salzberg et al. [32]	Retrospective study	*n*=863	Incidence of capsular contracture	Among 863 patients who underwent ADM-assisted implant reconstruction, 11.6% received radiotherapy. After a median follow-up of 4.7 years, only 1.9% of the irradiated breasts developed capsular contracture, and all cases occurred within the first 2 years.
	Caragher et al. [35]	Retrospective study	*n*=157	Complications	ADM was a protective factor against perioperative complications (OR=0.15, *P*=0.025), whereas no statistically significant association was found between ADM and radiotherapy-related complications.
	Lakhlani et al. [36]	Retrospective study	*n*=1234	Complications	In previously irradiated patients, no significant difference in overall complication rates was observed between the ADM and non-ADM groups; however, the use of ADM may reduce the likelihood of requiring image-guided drainage for hematoma or seroma (OR = 0.11, *p* = 0.039).
Implant position and radiotherapy	Sobti et al. [40]	Retrospective study	*n*=47	Incidence of capsular contracture	A significantly higher capsular contracture rate was observed in the subpectoral reconstruction cohort compared with the prepectoral reconstruction cohort (51.8% vs. 30.0%, *p* = 0.02).
	Sinnott et al. [41]	Retrospective study	*n*=66	Incidence of capsular contracture	The contracture rate was three times higher for the subpectoral reconstruction patients with PMRT than for the prepectoral reconstruction patients with PMRT (52.2 vs 16.1%; *p* = 0.0018).
	Shammas et al. [42]	Retrospective study	*n*=585	Incidence of capsular contracture	Under radiotherapy, the risk of capsular contracture was significantly higher in the subpectoral reconstruction group compared with the prepectoral reconstruction group (62% vs. 18%, *p* < 0.001)
Radiotherapy techniques	Kaidar-Person et al. [43]	Treatment planning study	NA	Dose	For targets with complex geometries, VMAT demonstrated superior performance in terms of both dose conformity and target volume coverage.
	Dumane et al. [44]	Prospective study	*n*=10	Dose	VMAT combined with DIBH significantly reduced cardiac and lung doses (self-controlled before-and-after comparison).
	Holt et al. [45]	Meta-analysis	13 studies (*n*=459)	Complications	Prosthetic implant removal (19%) was the most common complication of proton radiotherapy. However, conclusions were limited as only 5 studies focused on implant-based breast reconstruction.
	Naoum et al. [46]	Retrospective study	*n*=1617	Complications	When each modality group was compared with patients who did not receive radiotherapy, proton therapy was associated with a substantially greater likelihood of capsular contracture than photon therapy (OR=15.3∶2.5).
	De Sousa et al. [47]	Retrospective study	*n*=288	Complications	Compared with the no-bolus group (*n*=263), patients receiving bolus (*n*=25) demonstrated increased risks of expander infection (*P*=0.059) and expander loss (*P*=0.023), as well as a significant association with radiation dermatitis (*P*<0.001) during the first stage of reconstruction.
	Kim et al. [55]	Retrospective study	*n*=396	Complications	There was no significant difference in complication rates between conventional fractionation and hypofractionated radiotherapy. However, among patients who underwent immediate reconstruction, the incidence of severe capsular contracture was significantly lower in the hypofractionated radiotherapy group (*p* = 0.025).
	Song et al. [56]	Retrospective study	*n*=84	Complications	The complication rate in the hypofractionated VMAT radiotherapy group was lower than that in the conventional fractionated radiotherapy group (18.2% vs 44.8%, *p* = 0.012).
	Lin et al. [57]	Retrospective study	*n*=265	Complications	Univariate logistic regression revealed a significant association between boost irradiation and a higher incidence of complications, including hematoma and implant removal (OR: 2.199, *P*=0.017).
	Naoum et al. [58]	Retrospective study	*n*=746	Reconstruction failure, complications, local control	Compared with the non-boost group, chest wall boost was independently associated with significantly increased risks of implant exposure, definitive reconstruction failure, and overall implant loss (all *P* < 0.01), yet did not confer a significant benefit in preventing local recurrence (HR=1.16, *P* = 0.72).

*ADM* Acellular dermal matrix; *BIA-ALCL* Breast implant-associated anaplastic large cell lymphoma; *DIBH* Deep inspiration breath hold; *NA* Not applicable; *PMRT* Post-mastectomy radiation therapy.

## Methods

### Study Design

This study is a narrative review employing a systematic search strategy to ensure a comprehensive and transparent synthesis of the literature regarding implant-based breast reconstruction (IBBR) in the context of post-mastectomy radiation therapy (PMRT).

### Search Strategy

A comprehensive literature search was conducted across PubMed, Embase, and Web of Science for articles published up to April 2026. The search utilized a combination of Medical Subject Headings (MeSH) and free-text keywords, including “breast cancer,” “post-mastectomy radiation therapy (PMRT),” “implant-based breast reconstruction,” “acellular dermal matrix (ADM),” and “prepectoral reconstruction.” To ensure currency, recent high-impact studies and the latest consensus statements were manually retrieved and incorporated during the revision process.

### Inclusion and Exclusion Criteria

Studies were included if they met the following criteria: (1) Patients underwent mastectomy followed by implant-based reconstruction (one-stage or two-stage); (2) PMRT was administered; and (3) clinical outcomes were reported (e.g., capsular contracture, reconstruction failure, or other complications).

Exclusion criteria included: (1) purely autologous reconstruction; (2) case reports with fewer than five patients; and (3) studies with insufficient data for synthesis.

### Literature Screening and Data Synthesis

Titles and abstracts were screened for relevance, and full-text articles were assessed according to the predefined inclusion and exclusion criteria. A detailed flowchart of the literature screening process is provided in Supplementary Fig. 1.

### Timing of Implant-Based Reconstruction and Radiotherapy

The classification of implant-based breast reconstruction is based on two key factors: surgical timing and methodology, resulting in three categories: immediate, delayed, and delayed–immediate (two-stage) reconstruction.

#### Immediate versus Delayed Reconstruction

Immediate breast reconstruction (IBR) is conducted during the same operation as the mastectomy. Conversely, delayed breast reconstruction (DBR) is carried out in a subsequent procedure, after the completion of additional cancer therapies, such as chemotherapy or radiotherapy. Historically, IBR has been more widely adopted than DBR, primarily owing to lower surgical costs, a relatively low complication rate [10, 11], and the psychological benefit of avoiding a period without a breast mound.

High-quality studies directly comparing the safety of immediate versus delayed implant-based breast reconstruction remain scarce. Matar et al. [12] conducted a meta-analysis that initially included 30 studies comparing the outcomes of the two reconstructive approaches, yet only six of them solely focused on patients receiving PMRT. Based on these six studies, no statistically significant difference in complication rates was observed between immediate and delayed reconstruction. However, the generalizability of these findings is restricted by the inclusion of patients who did not receive implant-based reconstruction. Manyam et al. [13] compared complication rates in 77 patients who underwent IBR or DBR followed by radiotherapy. The results demonstrated that both the rate of complications requiring reoperation (38% vs 70%) and the reconstruction failure rate (22% vs 56%) were lower in the IBR group than in the DBR group. Notably, the failure rate decreased substantially after excluding failures attributable to infection, suggesting that meticulous infection control could significantly improve success rates. Although this study demonstrated certain advantages of immediate reconstruction in terms of complication rates and reconstructive outcomes, its conclusions were limited by the small sample size. The MD Anderson Cancer Center advocates that for patients with a preoperative indication for PMRT (e.g., Stage III breast cancer), delayed reconstruction may be preferable, as the reconstructed breast mound can present technical challenges for irradiating the internal mammary nodal chain [14]. Overall, the question remains controversial, and the existing data are insufficient to establish the superiority of either approach, particularly given that the evidence is predominantly derived from retrospective studies with inherent limitations, including selection bias and confounding variables. Further prospective studies are essential to identify the optimal strategy. Notably, infection is a critical contributor to reconstructive failure and thus warrants prophylactic measures in both approaches. Additionally, patients should be informed of radiotherapy-related risks, and clinical decisions should be individualized based on fully informed consent.

#### Delayed–Immediate (Two-Stage) Reconstruction

Delayed–immediate, or two-stage, reconstruction is particularly appropriate when the need for PMRT cannot be definitively determined prior to surgery [15].

This approach involves a two-stage process. The first stage consists of the immediate insertion of a tissue expander following mastectomy, while the second stage entails its replacement with a permanent implant, guided by postoperative pathological findings and the completion of adjuvant therapies. The optimal timing and sequencing of radiotherapy within this two-stage framework remain highly controversial. One approach involves irradiating the tissue expander, followed by replacement with a permanent implant (expander–radiotherapy–implant sequence). The alternative involves first performing the exchange, followed by irradiation of the permanent implant (expander–implant–radiotherapy sequence) [6]. The superiority of one sequence over the other has not been established. For patients whose expanders are irradiated, there are no explicit recommendations on the optimal interval before exchange surgery, although a delay of at least 6 months is common practice [16].

##### The Timing of Radiotherapy Intervention

The two-stage breast reconstruction and radiotherapy, when performed in different sequences, each has its own advantages and disadvantages. According to the accumulating literature, radiation directed at the tissue expander is associated with a reduced risk of capsular contracture. However, this benefit is counterbalanced by an increased reconstruction failure rate. Conversely, irradiating the permanent implant is related to a lower breast reconstruction failure rate but a higher incidence of capsular contracture [17–20]. A meta-analysis comparing complication rates and reconstruction failure rates between the two sequences, incorporated 11 studies (including 3 prospective controlled studies and 8 retrospective studies) involving 1,447 patients. The results indicated that the risk of capsular contracture in the irradiated tissue expander group was significantly lower (RR 0.47; 95% CI:0.29–0.78; *p*=0.003), and the incidence of other complications was comparable between the two groups. In contrast, the reconstruction failure rate was relatively lower in the irradiated permanent implant group (RR 1.71; 95% CI, 1.06–2.75; *p*=0.03) [21]. These findings further illustrate that each sequence has distinct merits and limitations.

The lower rate of capsular contracture in the expander irradiation group may be attributable to the opportunity for capsulotomy or capsulectomy during the implant exchange procedure following radiotherapy, which can address radiation-induced contracture and allow time for tissue recovery. The higher reconstruction failure rate is likely due to radiation-induced tissue fibrosis and adhesions, which complicate the subsequent surgery and impair wound healing, thereby compromising implant stability [20]. For patients receiving radiation to the permanent implant, the lower failure rate must be weighed against the elevated risk of capsular contracture. Whether the reduced risk of reconstruction failure justifies accepting a decline in aesthetic outcome warrants careful consideration.

Furthermore, the timing of radiotherapy intervention must also account for the potential impact of treatment delays caused by implant exchange on the tumor control. For patients with large primary tumors or node-positive disease, irradiating the permanent implant represents a less favorable sequence, as radiotherapy is deferred until after the exchange surgery [6]. Additionally, the inflation status of the tissue expander during radiation may influence outcomes. In a study by Woo et al., a cohort of 49 patients undergoing two-stage reconstruction received radiotherapy with the tissue expander in situ. The cohort was stratified into two groups based on expander status—deflated (*n*=20) and inflated (*n*=29)—to compare the incidence of adverse outcomes. Patients in the deflated group experienced significantly more complications overall (65% vs. 6.9%; *p*<0.001). Specific adverse events, namely hematoma (40% vs. 0%; *p*<0.001) and skin fibrosis/contracture (25% vs. 3.4%; *p*=0.035), were also markedly more prevalent in the deflated cohort [22]. Although long-term oncological outcomes were not assessed, this study highlights the importance of considering expander volume during treatment planning. Given the time-dependent nature of many complications and the scarcity of long-term follow-up data, there is an urgent need for prospective randomized trials to generate the high-level evidence required to establish the optimal protocol for PMRT in the context of two-stage implant reconstruction.

##### Optimal Timing for Expander–Implant Exchange After Radiotherapy

Peled et al. [16] investigated the optimal interval between the completion of radiotherapy and the expander-to-implant exchange surgery. In their study of 88 patients, 49 underwent exchange within 6 months of completing radiotherapy, while the remainder underwent exchange at ≥6 months. Patients in the <6-month cohort experienced nearly three times the incidence of implant removal (22.4%) compared with those in the ≥6-month group (7.7%), a difference that reached statistical significance (*P*=0.036). Despite the small sample size, this study provides preliminary guidance for clinical practice. Another study involving 34 patients who underwent implant-based reconstruction observed differences in radiotherapy-related complications based on the time interval to exchange. When a 4-month threshold was applied for group stratification, infection was more prevalent in the shorter-interval cohort (30% vs. 14.29%). In contrast, capsular contracture occurred more frequently in the longer-interval group (21.43% vs. 5.00%). However, these differences did not reach statistical significance (*p*=0.422 and *p*=0.283, respectively) [23]. The pathophysiology of radiation-induced tissue injury may explain these trends: Acute effects such as erythema and desquamation may predispose patients to infection when the interval is shorter, while late effects including tissue atrophy and fibrosis may contribute to capsular contracture with a longer interval. In current practice, most clinicians advocate an interval of ≥6 months. However, an individualized approach based on the resolution of skin and soft tissue injury may yield optimal outcomes.

## Reconstruction Materials and Radiotherapy

### Silicone and Saline Implants

The filler materials comprising contemporary breast implants mainly include silicone gels and physiological saline. Silicone implants are generally preferred for their more natural tactile quality and appearance, leading to higher patient satisfaction scores [24, 25], although they are more costly [26]. In the setting of radiotherapy, silicone implants may be susceptible to complications such as capsular contracture and implant rupture. By comparison, saline implants, filled with sterile saline solution, are theoretically less affected by radiation but provide a less natural feel and carry a risk of deflation. To date, no studies have directly compared the efficacy and safety of different implant fill materials in patients receiving PMRT, representing an important avenue for future investigation.

Implants are also classified by surface texture as either smooth or textured. Textured implants, characterized by a rough surface, can reduce the risk of capsular contracture [27] and enhance positional stability. However, subsequent evidence has established an association between textured implants and the development of breast implant-associated anaplastic large cell lymphoma (BIA-ALCL). A prospective cohort study at MSKCC followed 3,540 patients with textured implants. Within this cohort, ten patients were diagnosed with BIA-ALCL after a median implantation period of 11.5 years. The calculated incidence rate was 0.311 per 1,000 person-years (95% CI: 0.118–0.503) [28]. The underlying mechanism may involve the larger surface area of textured implants promoting increased bacterial adhesion and local inflammatory responses. Long-term chronic stimulation may drive lymphocyte activation, ultimately triggering malignant transformation and contributing to BIA-ALCL development [29, 30]. Future research should actively explore and rigorously compare the efficacy and safety of radiotherapy in patients with implants of different materials and textures. In clinical practice, clinicians should carefully weigh the benefits and risks associated with different implant types to optimize therapeutic outcomes.

### The Use of Acellular Dermal Matrix (ADM)

Adequate soft tissue coverage is paramount for successful implant-based reconstruction. To this end, acellular dermal matrix (ADM)—a biological mesh derived from human, porcine, or bovine sources—is increasingly utilized. The use of ADM offers several key advantages: It provides precise definition and structural support to the inframammary fold, effectively distributes tension on the mastectomy skin flaps, and ultimately yields superior aesthetic outcomes with a more natural contour.

For patients receiving PMRT, most studies support the use of ADM in implant-based reconstruction to potentially mitigate complications, particularly capsular contracture. Park et al. [31] compared implant-reconstruction patients receiving PMRT with ADM (*n*=15) versus without ADM (*n*=11). The incidence of infection (0% vs. 9.1%, *p*=0.42), wound dehiscence (6.7% vs. 9.1%, *p*=1), capsular contracture around the expander (33.3% vs. 63.6%, *p*=0.13), capsular contracture around the permanent implant (0% vs. 27.3%, *p*=0.06), and implant displacement (0% vs. 18.2%, *p*=0.17) were all lower in the ADM group. It should be noted that the observed trends did not reach statistical significance, a limitation likely attributable to the small sample size. Predictive factor analysis demonstrated a significant association between reconstruction type (with vs. without ADM) and the occurrence of complications (*p*=0.015). In a larger study by Salzberg et al. [32] involving 863 patients who underwent ADM-assisted implant reconstruction (11.6% of whom received radiotherapy), only 1.9% of irradiated breasts developed capsular contracture during a median follow-up of 4.7 years, with all cases occurring within the first 2 years. This suggests that ADM may genuinely reduce, rather than merely delay, the onset of capsular contracture. The proposed mechanisms include inhibition of myofibroblast differentiation, reduced vascularity, and decreased fibroblast proliferation within the implant capsule [31, 33], though further investigation is warranted. For patients concerned about post-PMRT capsular contracture, the addition of ADM is a viable consideration.

The role of ADM varies depending on the implant placement plane. In subpectoral reconstruction, ADM is primarily used to define the inframammary fold, whereas in prepectoral reconstruction, it serves as the principal soft tissue covering for the implant [34]. A key limitation of the current evidence is that studies evaluating irradiated patients have not stratified outcomes by implant plane. Caragher et al. [35] conducted a retrospective single-center study of 157 patients who underwent implant-based breast reconstruction and PMRT (prepectoral, *n* = 73; subpectoral, *n* = 84). Multivariable regression analysis identified ADM as a protective factor against perioperative complications (OR = 0.15, 95% CI: 0.03–0.78, *P* = 0.025), whereas no statistically significant association was observed between ADM use and radiotherapy-related complications (OR = 0.57, 95% CI: 0.05–7.11, *P* = 0.659). Another study evaluated complication rates following ADM-assisted implant reconstruction (prepectoral or subpectoral) in previously irradiated patients. The results revealed no significant difference in overall complication rates (including deformity and tissue necrosis) between the ADM and non-ADM groups. However, ADM use was associated with a lower likelihood of requiring image-guided drainage for hematoma or seroma (OR = 0.11, 95% CI: 0.01–0.89, *p* = 0.039) [36]. The differential role of ADM in prepectoral versus subpectoral reconstruction warrants further investigation.

### Implant Position and Radiotherapy

Implant-based breast reconstruction offers two primary surgical planes: prepectoral (anterior to the pectoralis major muscle) and subpectoral (posterior to the muscle). Initially popularized in the 1970s, the prepectoral approach was nearly abandoned owing to a high frequency of complications, most notably capsular contracture rates exceeding 50% [37]. With technical refinements, particularly the introduction of ADM, prepectoral breast reconstruction (PBR) has experienced a clinical resurgence. Contemporary evidence suggests that PBR in irradiated patients demonstrates a favorable safety profile, with overall complication, capsular contracture, and infection rates typically remaining below 20% [38, 39].

For patients requiring PMRT, PBR appears to represent a superior option compared with subpectoral breast reconstruction (SBR). Sobti et al. [40] compared post-radiation capsular contracture rates between SBR (*n*=27) and PBR (*n*=20) cohorts. A statistically significant difference (*p*=0.02) was observed between the two groups. The capsular contracture incidence in the SBR cohort reached 51.8% substantially exceeding the 30.0% recorded in the PBR group. No statistically significant differences were observed in other complications such as infections. Logistic regression confirmed that implant position was a significant predictor of capsular contracture (OR, 0.24; 95% CI, 0.08–0.64; *p*<0.01). A similar finding was reported by Sinnott et al. [41], in a study of 66 patients, wherein the capsular contracture incidence following PMRT with SBR was 3 times higher than that with PBR (52.2% vs. 16.1%; *p* = 0.0018). A single-center retrospective study including 585 patients further supported this finding, showing that under radiotherapy, the risk of capsular contracture was significantly higher in the SBR group compared with the PBR group (62% vs. 18%, *p* < 0.001) [42]. These findings suggest that PBR may confer greater radiation tolerance. The underlying mechanism may relate to radiation-induced fibrosis of the pectoralis major muscle in patients with subpectoral implant placement. Clinically, for patients with an indication for PMRT, PBR may be considered the preferred approach.

### Radiotherapy Techniques for Implant-Based Reconstruction

The decision to administer PMRT following reconstruction is based on the same clinical and pathological indications as for non-reconstructed patients. However, the presence of an implant necessitates special considerations, requiring careful balancing of implant-related complication risks with the technical demands of radiotherapy.

#### Radiotherapy Modalities

The choice of radiotherapy technique is critical for patients with implants. An international multicenter planning study compared 35 treatment plans for two implant-reconstruction cases using various techniques, including field-in-field intensity-modulated radiotherapy (FIF-IMRT), inverse planned IMRT, volumetric modulated arc therapy (VMAT), and helical tomotherapy [43]. For targets with complex geometries, VMAT demonstrated superior performance in terms of both dose conformity and target volume coverage. A limitation of VMAT is the potentially larger volume of low-dose radiation to surrounding organs at risk (OARs), which can be mitigated by combining VMAT with deep inspiration breath hold (DIBH). Dumane et al. [44] conducted a prospective study enrolling 10 patients who underwent implant-based reconstruction. A longitudinal comparison of OAR doses was performed for each patient before and after the combination of VMAT with DIBH. The prescribed dose was 50 Gy in 25 fractions. A significant reduction of 2.9 Gy was observed in the mean heart dose, declining from 8.2 to 5.3 Gy (*p*<0.05). The maximum dose to the left anterior descending (LAD) artery also decreased by 9.9 Gy (*p*<0.05). Furthermore, the V5 (volume receiving ≥5 Gy) was significantly reduced for all evaluated organs: a decrease of 29.6% for the heart, 5.8% for the ipsilateral lung, 15.4% for the contralateral lung, and 10.8% for the total lung volume (all *p*<0.05). This dose reduction is primarily attributable to the spatial separation achieved through DIBH. By expanding the thoracic cavity during breath hold, DIBH creates greater physical distance between critical structures—such as the heart and lungs—and the primary radiation field. This geometric advantage is effectively exploited by VMAT, which optimizes dose delivery to ensure high target uniformity while maximizing plan conformity. Consequently, the synergistic application of VMAT and DIBH is particularly advantageous for patients with implant-based reconstruction, as it significantly reduces the volume of OARs exposed to low-dose radiation.

Compared with photon radiotherapy, clinical experience with proton radiotherapy for implant-based reconstruction remains limited. In a meta-analysis by Holt et al. [45] evaluating the use of proton therapy for PMRT, 13 studies (459 patients) reported outcomes following reconstruction. The most frequently reported adverse event was implant removal (19%). However, only 5 of the included studies exclusively enrolled breast reconstruction patients, and not all involved implant-based cases. Consequently, the findings have certain limitations, making it difficult to accurately assess the efficacy of proton therapy specifically in the implant-based reconstruction population. In the study by Naoum et al. [46], a total of 1,617 patients were included, of whom 759 received PMRT. Among these, 8.3% underwent proton therapy, and the remainder received photon therapy. When each modality group was compared with patients who did not receive radiotherapy, proton therapy was associated with a substantially greater likelihood of capsular contracture than photon therapy (OR=15.3∶2.5). However, not all patients receiving PMRT had undergone implant-based reconstruction. Currently, the application of proton therapy in this clinical setting lacks a robust evidence base, and further research is needed.

#### Use of Bolus

Thinning of the skin and subcutaneous tissue can occur as a consequence of reconstructive surgery, potentially impairing blood supply to the overlying skin flap. The superficial anatomy of the chest wall presents a dosimetric challenge: The dose build-up region can result in inadequate skin dose, as the radiation dose does not reach its maximum immediately upon entering the body. To enhance the radiation dose delivered to superficial target volumes, a tissue-equivalent material known as bolus is often placed on the patient’s skin surface. To assess the effects of bolus use, a retrospective review was conducted on a cohort of 288 patients undergoing two-stage breast reconstruction, in whom radiotherapy was administered to the tissue expander [47]. Compared with the no-bolus group (*n*=263), patients receiving bolus (daily, *n*=6; alternate-day, *n*=19) demonstrated increased risks of expander infection (*P*=0.059) and expander loss (*P*=0.023), as well as a significant association with radiation dermatitis (*P*<0.001) during the first stage of reconstruction. Although the bolus groups were small, these findings warrant consideration. The European Society for Radiotherapy and Oncology (ESTRO) expert consensus states that for all post-mastectomy patients, regardless of reconstruction status, bolus application increases radiation-related skin toxicity. Nevertheless, bolus should be considered when there is significant risk of skin infiltration, particularly in situations where the 95–100% isodose curve fails to provide adequate coverage of the tumor bed [48]. In summary, despite its efficacy in augmenting superficial target dose, bolus application is associated with increased risks of infection and implant failure. Therefore, a judicious approach is recommended, limiting its use to cases in which the risk of skin recurrence is substantial.

#### Target Volume Delineation

Target volume delineation for PMRT in patients with implants must be individualized based on anatomy, disease stage, and pathological findings. In 2019, the ESTRO Advisory Committee in Radiation Oncology Practice (ACROP), published consensus guidelines defining the approach to target delineation in this population [49]. The recommendations vary by implant position:

- **Subpectoral Reconstruction:** For early-stage tumors with the implant in a subpectoral position, the chest wall clinical target volume (CTVcw) may be limited to the skin, subcutaneous tissue, and pectoralis major muscle. For tumors with documented invasion of the muscle or chest wall, the CTVcw may be expanded to include the implant and the underlying ribs and intercostal muscles.
- **Prepectoral Reconstruction:** The CTVcw should encompass the skin, subcutaneous tissue, the implant, and the pectoralis major muscle. In the presence of adverse pathological features or for deep-seated tumors, the target should also include the region not covered by the pectoralis muscle preoperatively, ideally guided by surgical clips marking the muscle’s original position.

Subsequently, a retrospective study by Joo et al. [50] clinically validated these target volume delineation guidelines. This study included 51 patients with implant-based reconstruction (encompassing 65 recurrent lesions) from four institutions and performed an in-depth analysis of the relationship recurrence locations and the ESTRO-defined target volumes. The results demonstrated that the ESTRO target volumes provided coverage of 93%–100% of recurrent lesions, with an overall coverage rate of 94.0%. Specifically, the coverage rate for subpectoral implants was 93.0%, while that for prepectoral implants reached 100%. These findings strongly support the clinical validity and applicability of the ESTRO target volume consensus guidelines.

However, these guidelines, developed based on a predominantly European population, may not be fully generalizable to Asian patients owing to anatomical differences. The 2022 guidelines from China’s National Cancer Center also provide recommendations stratified by implant position but lack the nuanced approach for “adverse features” incorporated in the ESTRO guidelines [51].

Neither guideline offers specific protocols for individualizing target volumes in cases of multifocal tumors, extranodal extension, or post-neoadjuvant chemotherapy downstaging. Furthermore, both lack follow-up data beyond 10 years to assess long-term risks, such as secondary malignancies.

#### Dose Fractionation

Conventional fractionation (45.0–50.4 Gy in 25–28 fractions) is the most established regimen for PMRT. However, recent evidence suggests that hypofractionated radiotherapy is an acceptable alternative for patients with reconstruction [52–54]. A retrospective study from South Korea enrolled 396 patients who underwent breast reconstruction, of whom 267 received immediate reconstruction (91 with conventional fractionation and 176 with hypofractionation), and 129 received delayed reconstruction (48 with conventional fractionation and 81 with hypofractionation) [55]. The researchers compared the incidence of any breast complications (such as hematoma, wound infection, and capsular contracture) and major breast complications (any complication requiring reoperation or rehospitalization) between conventional fractionation (1.82 Gy per fraction) and hypofractionation (2.4–2.7 Gy per fraction) within each reconstruction subgroup. No statistically significant differences were observed in the immediate reconstruction cohort when comparing conventional and hypofractionated regimens, either for overall breast complications (*p*=0.093) or for major complications (*p*=0.814). Notably, compared with conventional fractionation, the rate of severe capsular contracture reported among patients receiving hypofractionated radiotherapy was significantly lower (*P*=0.025). Similarly, in the delayed reconstruction group, no statistically significant differences were observed in the incidence of any breast complications (*p*=0.129) or major breast complications (*p*=0.623) between the two fractionation regimens. However, this study also included some patients with autologous reconstruction. Another retrospective study by Song et al. [56] compared patients receiving conventional 3D-CRT (*n*=29; 50.4 Gy in 28 fractions) with those receiving hypofractionated VMAT (*n*=55; 40.05 Gy in 15 fractions). The incidence of radiation-related complications was 18.2% in the hypofractionated group, substantially lower than the 44.8% rate in the conventional fractionation group, a difference that reached statistical significance (*p*=0.012). Two prospective, randomized phase III trials (NCT03414970 and NCT03422003) are currently underway to provide high-level evidence on this topic.

Theoretically, a radiation boost to high-risk areas of the chest wall could improve local control; however, multiple studies indicate that it may increase complication rates following reconstruction. In the retrospective analysis by Lin et al., a cohort of 265 patients receiving PMRT was studied, of whom 146 received additional boost irradiation targeting the chest wall or surgical incision [57]. Univariate logistic regression revealed a significant association between boost irradiation and a higher incidence of complications, including hematoma and implant removal (OR: 2.199, 95% CI: 1.154–4.188, *P*=0.017). What is more, a retrospective analysis by Naoum et al. [58] examined the effects of chest wall boost irradiation in patients undergoing PMRT with breast reconstruction. The study involved 746 patients, with outcomes focusing on reconstruction success and local control. Among these, 559 patients (75%) underwent implant-based reconstruction. Within this subgroup, 275 patients (49%) received a chest wall boost (10 Gy in 5 fractions), while 284 patients (51%) did not. Multivariate analysis demonstrated that chest wall boost administration was independently associated with significantly increased risks of implant exposure, definitive reconstruction failure, and overall implant loss (corresponding odds ratios: 4.02, 3.15, and 2.04, all *P* < 0.01). Regarding local control, the 5-year local recurrence rate was 2.9% (95% CI: 1.5%–5.6%) in the non-boost group, compared with 4.4% (95% CI: 2.7%–7.2%) in the boost group. Statistical analysis indicated that chest wall boost irradiation did not confer a significant benefit in preventing local recurrence (HR=1.16, *P* = 0.72). Based on these findings, a cautious approach is recommended when considering chest wall boost irradiation. Further studies are needed to identify specific patient subgroups that may derive clinical benefit from this treatment strategy.

## Limitations

This review has several limitations. First, the majority of the included studies were retrospective in design, with a paucity of prospective trials, leading to inherent methodological limitations such as selection bias and confounding variables. Second, some studies had small sample sizes, which may compromise the stability and generalizability of the results. Additionally, head-to-head comparative studies were lacking in certain areas, necessitating further investigation.

## Summary and Future Directions

This review provides a comprehensive overview of the interdisciplinary field of implant-based breast reconstruction and radiotherapy. It extends beyond general discussions regarding whether or when to administer radiotherapy, delving into the technical aspects of how to optimize radiotherapy delivery. Furthermore, it offers clinicians a synthesis of the latest evidence and decision-making support to address challenges encountered in clinical practice.

Based on the available evidence and our interpretation, several practical recommendations can be proposed. The timing of reconstruction should be individualized according to the anticipated need for PMRT. In patients with a clear indication for PMRT and high-risk features, a more cautious approach is warranted, and delayed–immediate reconstruction may be considered. Within the two-stage reconstruction, the sequencing of radiotherapy requires careful balancing: Irradiation of the tissue expander may reduce the risk of capsular contracture but may increase reconstruction failure, whereas irradiation of the implant shows the opposite trend. Surgical strategies should also be individualized, as prepectoral reconstruction and the use of acellular dermal matrix may help mitigate radiotherapy-related complications. From a radiotherapy perspective, advanced techniques such as VMAT combined with DIBH may be preferred, when feasible, to optimize dose distribution and minimize exposure to organs at risk. The use of bolus and chest wall boost should be applied cautiously, with careful consideration of potential benefits and risks.

The management of breast cancer reconstruction and radiotherapy must ultimately balance long-term oncological control with the final aesthetic outcome. These goals are best achieved through a multidisciplinary team that comprehensively evaluates the patient’s tumor characteristics, physical condition, and personal preferences. An individualized approach is paramount to minimizing tumor recurrence and reconstruction complications while maximizing patient satisfaction and quality of life.

## Supplementary Information

Below is the link to the electronic supplementary material.

Supplementary file1 (JPG 58 KB)

## Declarations

### Human or Animal Participation

This article does not contain any studies with human participants or animals performed by any of the authors.
